# Using stable isotopes to trace sources and formation processes of sulfate aerosols from Beijing, China

**DOI:** 10.1038/srep29958

**Published:** 2016-07-20

**Authors:** Xiaokun Han, Qingjun Guo, Congqiang Liu, Pingqing Fu, Harald Strauss, Junxing Yang, Jian Hu, Lianfang Wei, Hong Ren, Marc Peters, Rongfei Wei, Liyan Tian

**Affiliations:** 1 Center for Environmental Remediation, Institute of Geographic Sciences and Natural Resources Research, Chinese Academy of Sciences, Beijing 100101, China; 2 University of Chinese Academy of Sciences, Beijing 100049, China; 3 State Key Laboratory of Environmental Geochemistry, Institute of Geochemistry, Chinese Academy of Sciences, Guiyang Guizhou 550002, PR China; 4 State Key Laboratory of Atmospheric Boundary Layer Physics and Atmospheric Chemistry, Institute of Atmospheric Physics, Chinese Academy of Sciences, Beijing 100029, China; 5 Institut für Geologie und Paläontologie, Westfälische Wilhelms-Universität Münster, Corrensstrasse 24, 48149 Münster, Germany

## Abstract

Particulate pollution from anthropogenic and natural sources is a severe problem in China. Sulfur and oxygen isotopes of aerosol sulfate (δ^34^S_sulfate_ and δ^18^O_sulfate_) and water-soluble ions in aerosols collected from 2012 to 2014 in Beijing are being utilized to identify their sources and assess seasonal trends. The mean δ^34^S value of aerosol sulfate is similar to that of coal from North China, indicating that coal combustion is a significant contributor to atmospheric sulfate. The δ^34^S_sulfate_ and δ^18^O_sulfate_ values are positively correlated and display an obvious seasonality (high in winter and low in summer). Although an influence of meteorological conditions to this seasonality in isotopic composition cannot be ruled out, the isotopic evidence suggests that the observed seasonality reflects temporal variations in the two main contributions to Beijing aerosol sulfate, notably biogenic sulfur emissions in the summer and the increasing coal consumption in winter. Our results clearly reveal that a reduction in the use of fossil fuels and the application of desulfurization technology will be important for effectively reducing sulfur emissions to the Beijing atmosphere.

In recent years, most parts of northern and eastern China have been strongly affected by haze events, arousing public and official concerns^1^,^2^. Haze results from a high concentration of submicron (10–100 nm) particles with high scattering coefficients and high relative humidity in the atmosphere tends to aggravate these effects, which may cause visibility reduction to 3–4 km^3^. Sulfate serving as cloud condensation nuclei^4^,^5^ is an important component of atmospheric aerosols^6^. Such sulfate aerosols influence the surface temperature of the Earth^7^, acid rain formation^8^,^9^, and human health^10^, playing a key role in environmental chemistry and climate change^6^,^11^,^12^. Therefore, knowing the source(s) of aerosol sulfate and its transport and transformation in the atmosphere will form the necessary base for improving the air quality in Beijing.

Stable isotopes of sulfur and oxygen are used for identifying potential sources of atmospheric sulfate^13^,^14^,^15^,^16^. The sulfur isotope ratios (δ^34^S) of sea salt sulfate, dimethyl sulfide (DMS) and anthropogenic sulfate are +21‰^17^, +18.9 to +20.3‰^18^ and +1 to +11‰^19^,^20^, respectively. However, the δ^34^S values of biogenic sulfur released from soils and wetlands are much lighter, ranging from −10 to −2‰^21^,^22^,^23^. The relative contributions for each source of sulfate can be calculated according to their distinct δ^34^S values^16^,^20^. Moreover, if sources of sulfate cannot be distinguished due to their similar sulfur isotope values, the oxygen isotopic composition of sulfate (δ^18^O_sulfate_) can provide additional evidence^24^. δ^18^O_sulfate_ is influenced by source variation and mixing^25^. Hence, paired sulfur and oxygen isotopes a powerful tool for constraining the source of sulfate^24^,^26^.

Sulfur and oxygen isotopes have also been used to investigate the oxidation processes of SO_2_ and transport pathways of sulfur in the atmosphere^14^,^27^,^28^. Sulfur isotopes show distinctive isotope fractionations for different oxidation reactions of SO_2_^28^,^29^,^30^,^31^. Sulfur isotope enrichment in sulfate can be caused by heterogeneous oxidation of SO_2_^29^,^30^, while sulfur isotope depletion in sulfate may be generated from homogeneous oxidation^31^. However, the sulfur isotope fractionation factor α = 1.14 for homogeneous oxidation has been estimated based on RRKM transition-state theory^32^. Furthermore, a recent study shows that only transition metal-catalysed oxidation of SO_2_ can lead to the enrichment of lighter sulfur isotopes in sulfate, compared with oxidation by OH, H_2_O_2_ and O_3_^28^. In addition, the oxygen isotope composition of sulfate may reflect oxidation processes^27^, since oxygen isotopes of sulfate and water are not exchanged under ambient conditions^25^. Primary sulfate formed in the emission source may be enriched in heavy oxygen isotope (δ^18^O > +20‰)^33^,^34^, while the δ^18^O values of secondary sulfate range from ~−10‰ to +20‰^25^. The proportion of primary and secondary sulfate can be estimated based on an oxygen isotope apportionment model^14^.

Seasonal variations in sulfur and oxygen isotopes of atmospheric sulfate and SO_2_ have been observed^25^,^35^,^36^,^37^,^38^. The sulfur isotope ratios often show low values in summer and high values in winter^13^,^35^,^37^, while it displays an opposite seasonal tendency in Central Europe^38^. The seasonality of sulfur isotopes is attributed to several possible factors, including seasonal changes of the sources of atmospheric sulfur^35^,^38^, seasonal variations in the proportion of oxidation pathways^37^,^39^, seasonal changes in the isotope fractionation factors due to temperature-dependence^36^ and seasonality in reservoir effects^39^. The δ^18^O value of sulfate in precipitation also shows seasonal variations, which could be related to changes in the oxygen isotopic composition of local precipitation^25^.

In this work, the sources of sulfate aerosols in Beijing and their formation, migration and transformation are studied by using stable sulfur and oxygen isotopes, which may provide a theoretical basis for air quality improvement in the city of Beijing. Furthermore, the seasonality in sulfur and oxygen isotopes of sulfate aerosols is discussed in order to better understand isotope fractionation during the oxidation of SO_2_.

## Results

### Water-soluble ions

**Figure 1:**
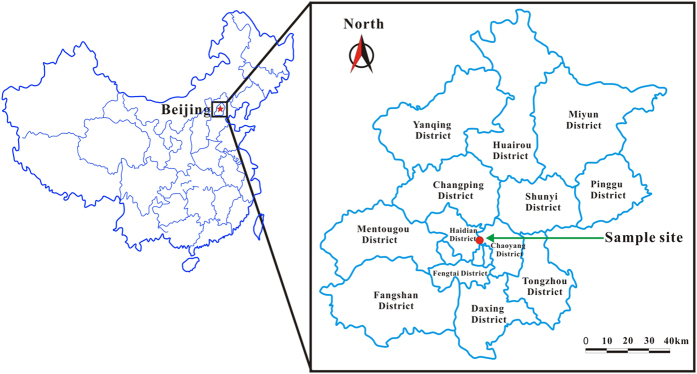
Sampling location in Beijing, China. Total suspended particulates (TSP) were sampled on a 3-day basis from May 31, 2012 to Jun 10, 2014 (n = 73) at the roof of a building (around 10 meters above ground level) in the site. Modified after Guo *et al*. (2013)^56^. Reprinted from Environmental Pollution, 176(2013), Guo *et al*., Tracing the source of Beijing soil organic carbon: A carbon isotope approach, 208–214, Copyright (2013), with permission from Elsevier.

**Figure 2:**
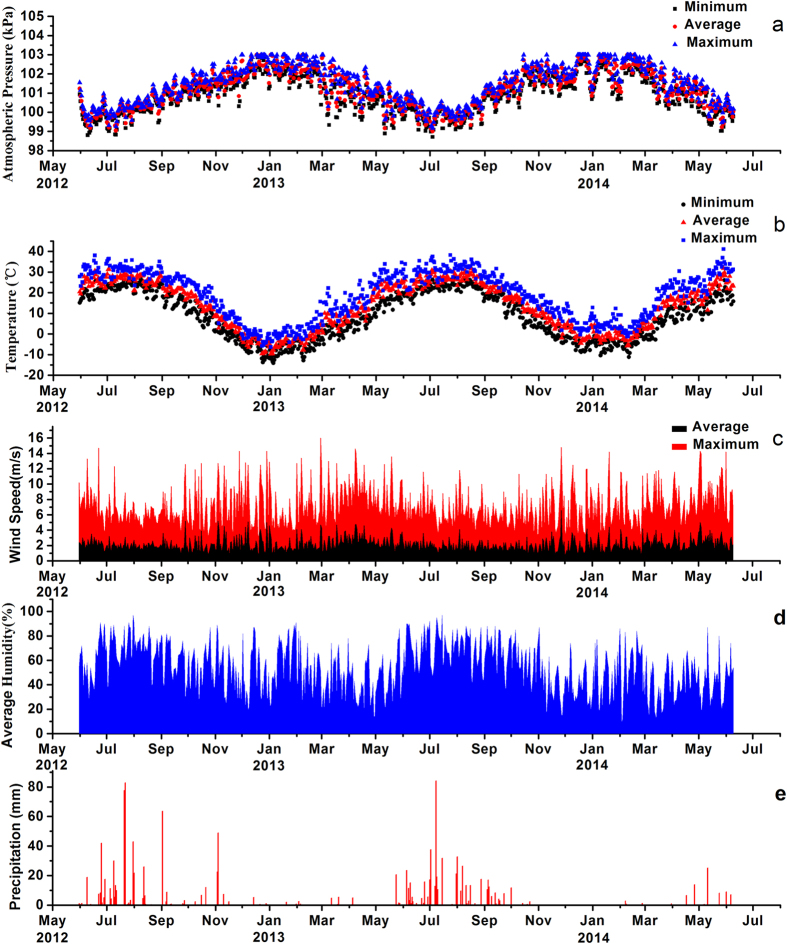
Meteorological parameters from May 2012 to June 2014 in Beijing, China (data from China Meteorological Data Network^57^).

**Figure 3:**
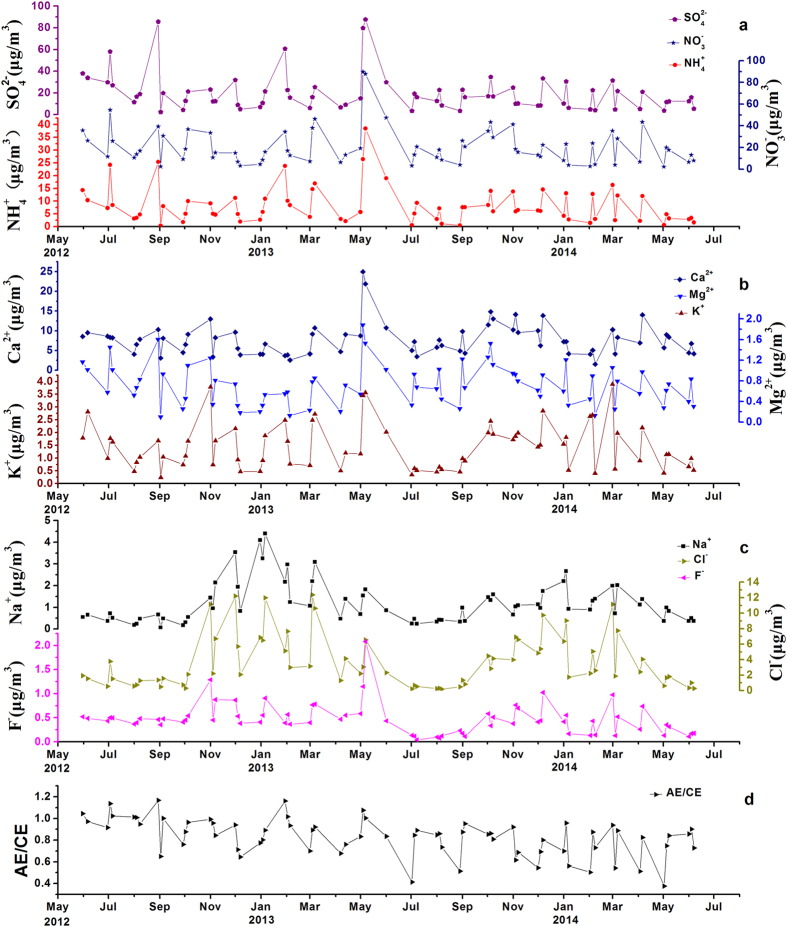
Variations in concentrations of water-soluble ions and ratios of the anion equivalents (AE) to the cation equivalents (CE) in atmospheric aerosols from May 2012 to June 2014 in Beijing, China.

Total suspended particulates (TSP) were sampled on a 3-day basis from May 31, 2012 to June 10, 2014 (n = 73) in Beijing (Fig. 1). Figure 2 shows the meteorological data during the sampling. The concentrations of water-soluble inorganic ions (WSII) in the TSP during the sampling period are shown in Table 1 and Fig. 3. The predominant ions in the TSP were 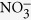, 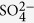, 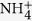 and Ca^2+^, which together account for ~85% of WSII. Nitrate and sulfate are the dominant anions, varying from 2.3 to 89.8 μg/m^3^ (mean = 21.1 ± 17.5 μg/m^3^, n = 70) and from 2.4 to 87.7 μg/m^3^ (mean = 20.0 ± 18.0 μg/m^3^, n = 70), respectively; while the concentrations of 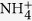 and Ca^2+^ range from 0.3 to 38.5 μg/m^3^ (mean = 8.3 ± 7.2 μg/m^3^, n = 70) and from 1.5 to 25.0 μg/m^3^ (mean = 7.8 ± 4.1 μg/m^3^, n = 70), respectively. The average concentrations of F^−^, Na^+^, K^+^ and Mg^2+^ are lower than 2.0 μg/m^3^.

The concentrations of WSII show seasonal changes (Fig. 3). 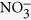, F^−^ and Ca^2+^ concentrations are relatively high in spring and autumn compared to summer and winter. The 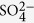 concentration is slightly lower in winter than in summer. The concentrations of Cl^−^ and Na^+^ are much higher in winter than in summer, spring and autumn.

The ion balance as an indicator of the acidity of the aerosols was calculated using the ratios of the anion equivalents (AE) to the cation equivalents (CE) in TSP samples^2^ (Fig. 3d). The ratios of AE/CE during the sampling period range from 0.38 to1.17 with a mean value of 0.83 ± 0.17 (n = 70). Most of ratios of AE/CE are lower than 1.0, which shows no seasonal changes.

### Sulfur and oxygen isotopes

**Figure 4:**
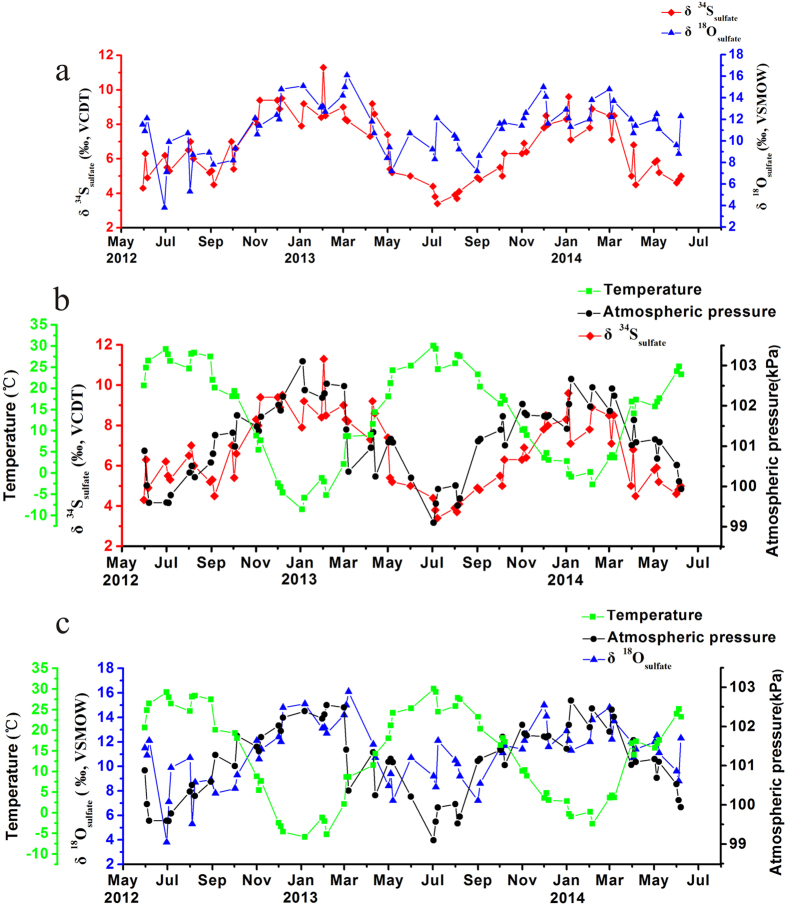
Variations in δ^34^S_sulfate_ and δ^18^O_sulfate_ values of atmospheric aerosols from May 2012 to June 2014 in Beijing, China and their relationship with the mean temperature (72 h) and mean atmospheric pressure (72 h).

Sulfur and oxygen isotopic compositions of sulfate (δ^34^S_sulfate_ and δ^18^O_sulfate_) in Beijing aerosols are presented in Fig. 4a. The δ^34^S_sulfate_ values range from 3.4 to 11.3‰ with a mean value of 6.6 ± 1.8‰ (n = 70). The δ^18^O_sulfate_ values vary from 3.8 to 16.1‰ around a mean value of 11.1 ± 2.4‰ (n = 66). Both isotope records reveal seasonal changes, i.e., low values in summer and high values in winter (Fig. 4a). The average values of δ^34^S_sulfate_ in spring, summer, autumn and winter are 6.4 ± 1.5‰ (n = 12), 5.0 ± 0.9‰ (n = 24), 6.8 ± 1.3‰ (n = 12) and 8.6 ± 0.9‰ (n = 22), respectively. The mean values of δ^18^O_sulfate_ are 10.6 ± 1.7‰ (n = 11), 9.3 ± 2.1‰ (n = 23), 11.1 ± 1.3‰ (n = 11) and 13.4 ± 1.4‰ (n = 21) for spring, summer, autumn and winter, respectively.

## Discussion

**Figure 5:**
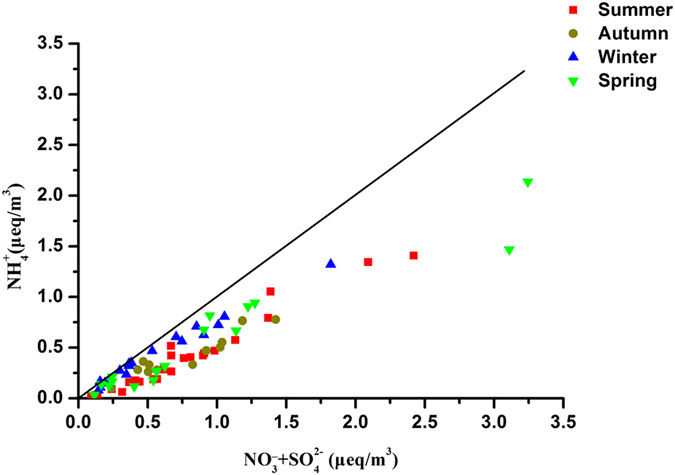
Ammonium equivalent concentration as a function of sum of the sulfate and nitrate equivalent concentrations in TSP from Beijing.

In order to determine the relationship between ions in TSP (as well as the sulfur and oxygen isotopic compositions), correlation coefficients are calculated (Table 2). A strong correlation is observable between 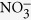 and 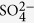 (r = 0.83), implying the similar chemical behavior in cloud processes^40^. 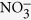 and 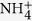 (r = 0.90) as well as 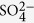
and 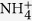 (r = 0.93) show strong correlations, indicating that NH_4_NO_3_ and (NH_4_)_2_SO_4_ may be two main species in TSP. At lower concentrations, the ratio of 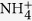
to 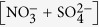 almost equals 1.0 (Fig. 5), which implies an ammonium-rich environment for these samples. However, the ratio of 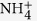
to 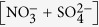 is lower than 1.0 at increasing 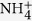 concentrations, suggesting that nitrate may exist in other chemical forms besides NH_4_NO_3_. A significant correlation is also found between Ca^2+^ and Mg^2+^ (r = 0.81), which may be an indicator of terrestrial sources (e.g. soil and miral dust). In addition, strong correlations between Ca^2+^ and 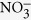 (r = 0.81) as well as Mg^2+^ and 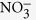
(r = 0.84) indicate heterogeneous chemistry on mineral dust^41^.

The relative importance of mobile versus stationary sources of nitrogen and sulfur in the atmosphere can be indicated by the mass ratio of 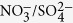
42,43. A high 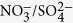 ratio implies that mobile sources of the pollutants are predominant over stationary sources^42^. However, the majority of the ratios are lower than 0.8 during the heating period, suggesting the predominance of stationary sources (emission from coal combustion) over mobile sources of pollutants.

The Cl^−^ and Na^+^ concentrations are positively correlated (r = 0.83) and display an increase in winter (Fig. 3c), suggesting they have a common source. As the prevailing winds in Beijing’s winter are north and northwest, a significant contribution from seawater in Beijing aerosols can be ruled out. In addition, the ratio of Cl^−^ to Na^+^ in winter is 3.2 ± 1.2, which is different from the ratio in seawater of 1.17^43^. Studies reported that high Cl^−^ concentration in Beijing aerosols may be related to coal combustion^2^,^43^. During combustion, complex changes in coal particles may cause the vaporization of volatile elements, including sodium^44^. Sodium vaporised from coal during combustion, may be present in the gas phase or bound in particulate aerosols in the flue gases, which can be emitted to the atmosphere^44^. Significant correlations exist as well for δ^34^S and Cl^−^ (r = 0.67) and for δ^34^S and Na^+^ (r = 0.66), which provides additional evidence for a common origin and the significant contribution of coal combustion to the atmospheric sulfate pool.

**Figure 6:**
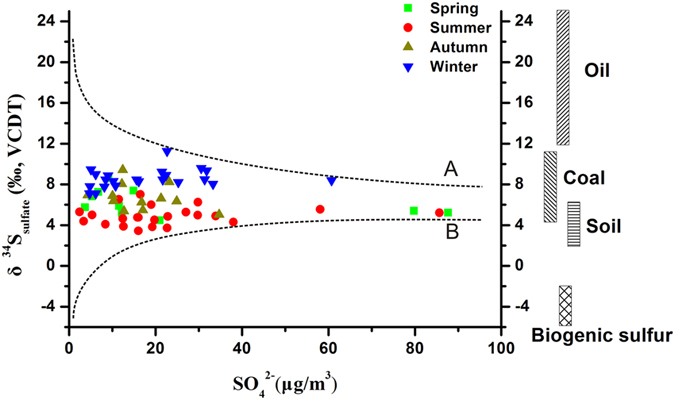
δ^34^S_sulfate_ and sulfate concentration in aerosols of Beijing compared to a ternary mixing model. Curve A represents a mixture between sulfur in oil with a δ^34^S value of 20.5‰ and sulfur in coal with a δ^34^S value of 6.6‰; Curve B represents a mixture between biogenic sulfur with a δ^34^S value of −10‰ and sulfur in coal with a δ^34^S value of 6.6‰.

Water-soluble sulfate in aerosol is derived from both primary (e.g. sea salt, dust, fly ash) and secondary (e.g. oxidation of SO_2_ and H_2_S) sulfates^14^,^37^, all characterized by their own distinct isotopic composition. Consequently, the sulfur isotopic composition of sulfate in Beijing aerosols reveals a mixture from different sulfate sources with high and low δ^34^S values (Fig. 6). Volcanism as a source of sulfate in Beijing aerosols can be excluded with no volcanic activities in North China^45^. Also, a significant contribution from sea salt is not very likely as suggested by the low concentration of Na^+^ (mean = 1.2 ± 1.0 μg/m^3^, n = 70). In addition, the weak correlation between 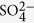 and Na^+^ (r = 0.16) as well as 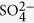 and Cl^−^(r = 0.19) also suggest that seawater sulfate provides only a very small contribution to the aerosols in Beijing.

Some SO_2_ emissions from industry and transportation ultimately originate from oil combustion. It has been estimated that 15.3 million tons of petroleum products were consumed in Beijing in 2012, including 4.4 million tons by industry and 6.1 million tons from transportation^46^. The sulfur content in oil from North China ranges from 0.1% to 0.6% and its δ^34^S value varies between 13.7‰ and 24.2‰ (mean = 20.5‰, n = 4)^47^. The emission rate of SO_2_ from oil combustion is relatively constant with almost no seasonal change in the consumption of petroleum. Therefore, the SO_2_ emissions from oil combustion in the study area are a steady source of sulfate in the aerosol that is characterized by a relatively high δ^34^S value.

The contribution from coal combustion to the atmospheric sulfur pool of China is very significant^15^,^37^. In Beijing, the total consumption of coal was 22.7 million tons in 2012 based on the China Energy Statistical Yearbook (2013)^46^. With an average sulfur content of 0.77wt.% in coal from North China^48^, an estimated 174.8 thousand tons of sulfur were released into Beijing’s atmosphere in 2012 assuming that there is no desulfurization implemented into the coal combustion processes. During the winter, the consumption of coal in China is increasing due to heating, which will affect the overall δ^34^S value of atmospheric sulfate. It has been shown before that the sulfur isotopic composition of atmospheric sulfur in the different regions of China is closely related to the sulfur isotope signature of the coal used in the respective area^37^. Reported δ^34^S values for coal from North China (mean = +6.6‰) are higher than for coal from South China (mean = −0.32‰)^47^,^48^. The average δ^34^S value of sulfate in aerosol from Beijing (6.6 ± 1.8‰, n = 70), determined in this study, is similar to that for coal used in North China, indicating that coal combustion is a significant, if not the most important contributor to the atmospheric sulfate pool.

Biogenic sulfur from wetlands and soils is an important source for atmospheric sulfate, especially in the summer^22^,^23^, and δ^34^S values for biogenic sulfur are generally negative, ranging from −10 to −2‰^21^,^22^,^23^. Low δ^34^S values recorded for Beijing summer aerosols may, thus, reflect a larger contribution from biogenic sulfur, in contrast to the winter season where low temperatures greatly attenuate (or inhibit) microbial activity in wetlands and soils.

Sulfates in aerosols can also originate from terrigenous sources (e.g. soil or mineral dusts). In this study, a significant correlation can be seen between 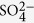 and Ca^2+^ concentrations (r = 0.63), which suggests a terrigenous contribution to the aerosol sulfate pool. Ca^2+^ as a reference element for mineral dust is used for calculating the proportion of this contribution (f_ts_) to sulfate in the aerosol by following the equation^49^:


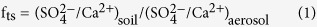


where the ratio of 
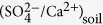
 is 0.18^50^.

The δ^34^S values of sulfate in soils from North China vary from 2.0 to 7.0‰^51^,^52^.

Assuming that the seasonal change in the δ^34^S values of aerosol sulfate reflects temporal variations in the proportional ctributions from different sulfate sources, these proportions can be estimated using the following equations^16^,^20^:


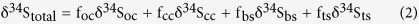



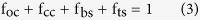


where f_oc_, f_cc_, f_bs_ and f_ts_ represent the fractional contributions of oil combustion, coal combustion, biogenic source and terrigenous source, respectively, and δ^34^S_oc_, δ^34^S_cc_, δ^34^S_bs_ and δ^34^S_ts_ represent the corresponding δ^34^S value of each sulfur source.

In winter, biogenic sulfur is likely negligible since the soil microbial activity is weak. Hence, we assume that in winter, f_bs_ equals 0. In addition, the contribution of oil combustion is relatively constant throughout the year as there is no seasonal variation in oil consumption. By solving equations (1)–(3), 34, 35, the contributions of sulfate sources can be calculated (results listed in Table 3), assuming a δ^34^S_oc_ value of 20.5 ± 4.8‰^47^, a δ^34^S_cc_ value of 6.6 ± 3‰^47^,^48^, a δ^34^S_bs_ value of −6 ± 4‰^21^,^22^,^23^ and a δ^34^S_ts_ value of 4.5 ± 3.5‰^51^,^52^ as the respective δ^34^S signature of each sulfur source. The results show that the average contributions of coal combustion, oil combustion, biogenic sulfur and terrigenous sulfate to sulfate in aerosols of Beijing are 49.6 ± 7.5%, 17.6 ± 8.6%, 19.8 ± 9.9% and 10.1 ± 6.2%, respectively, but exhibiting strong seasonal differences (Table 3).

It has been shown that the seasonal change in the proportion of different oxidation pathways of atmospheric sulfur dioxide to aerosol sulfate may also lead to a seasonality in δ^34^S_sulfate_^37^,^39^. The sulfur isotope fractionation factors (α) for different oxidation reactions of SO_2_ are distinct to each other^28^,^29^,^30^,^31^. Experimental studies show that the fractionation factor for heterogeneous oxidation is 1.0165 ± 0.001 at 25 °C^29^,^30^. The fractionation factor during gas-phase oxidation by the OH radical (homogeneous oxidation) is 0.991, which is determined by using an ab initio quantum mechanical calculation^31^. In contrast, results from laboratory measurements show that the fractionation factors during homogeneous oxidation and aqueous oxidation by H_2_O_2_ and O_3_ are greater than 1.0, while the fractionation factor for oxidation by transition metal ion catalysis (TMI-catalysis) is α_Fe_ = 0.9894 ± 0.0043 at 19 °C^28^. A recent study shows that the changing proportion among oxidation by TMI-catalysis, OH and H_2_O_2_ was the main cause for the seasonality in the δ^34^S values of sulfate versus SO_2_^39^. However, sulfate from aqueous SO_2_ oxidation by TMI-catalysis only accounts for 9–17% of the global sulfate production^53^. Thus, it cannot resolve the difference in δ^34^S_sulfate_ observed between summer and winter in Beijing aerosol sulfate.

A strong negative correlation between mean air temperature and δ^34^S_sulfate_ in aerosol (r = −0.83, Fig. 4b) is apparent. This could indicate that the seasonality in δ^34^S of atmospheric sulfate may result from a seasonal variation in sulfur isotope fractionation factors influenced by temperature^36^,^54^. Caron *et al*. revealed that during the oxidation of SO_2_ to sulfate, the δ^34^S_sulfate_ value increases by 0.08–0.15‰ with a decrease in temperature of 1 °C^54^. Considering a temperature difference of 30 °C between summer and winter in Beijing, this effect may, thus, cause a seasonal variation in δ^34^S_sulfate_ of 2.4 to 4.35‰. The maximum difference between the δ^34^S values in summer and winter, however, is 7.9‰, which is substantially higher. Consequently, the temperature effect alone cannot explain the seasonal difference in δ^34^S. In addition, a recent study shows that for the oxidation of SO_2_ by OH radicals, H_2_O_2_ and transition metal ion catalysis (TMI-catalysis), the coefficients of the temperature effect on the fractionation factors are 0.004 ± 0.015‰°C^1−^, 0.085 ± 0.004‰°C^1−^ and 0.237 ± 0.004‰°C^1−^, respectively^39^. This indicates that the temperature effect on the fractionation factor is negligible for the OH radical pathway, but can be more significant for the oxidation by TMI-catalysis and H_2_O_2_^39^. A temperature difference of 30 °C between summer and winter could account for a maximum seasonal isotopic difference via TMI-catalysis of 1.2‰, again insufficient for the observed maximal seasonality in δ^34^S_sulfate_.

In addition to temperature, a positive correlation between atmospheric pressure and δ^34^S_sulfate_ values can be observed (r = 0.69, Fig. 4b). Leung *et al*. evaluated the sulfur isotope fractionation factor (α) during the oxidation of SO_2_ by OH radicals based on the RRKM transition-state theory, and found that the factor is a function of pressure and temperature, i.e., α = 1.1646 + 0.0198(P/Torr)^0.1769^ −0.3092[(T/K)/1000]^32^. The maximum difference in atmospheric pressure between summer and winter is around 4 kpa (30 Torr), which may cause a change in the δ^34^S_sulfate_ value by 0.45‰ for the OH oxidation of SO_2_. It suggests that the change in atmospheric pressure may have only a minor effect on the variation in δ^34^S_sulfate_.

Considering that changes in the sulfur isotopic fractionation resulting from meteorological boundary conditions (i.e. air temperature and atmospheric pressure) are insufficient to explain the observed seasonality in δ^34^S_sulfate_, respective variations are more likely reflecting temporal changes in the proportional contributions from different sulfate sources during different times of the year, as has been reported from other areas before^38^. It is proposed here that during the summer, biogenic sulfur emissions which are characterized by negative δ^34^S values, are an important source of atmospheric sulfate^22^,^23^, leading a decrease in the overall δ^34^S value of aerosol sulfate in the summer. In contrast, the increase in coal consumption for heating during winter time (and with it an increase in the proportional importance of this contribution to the overall sulfate pool) will cause a shift to a more positive overall δ^34^S value for aerosol sulfate in the winter.

**Figure 7:**
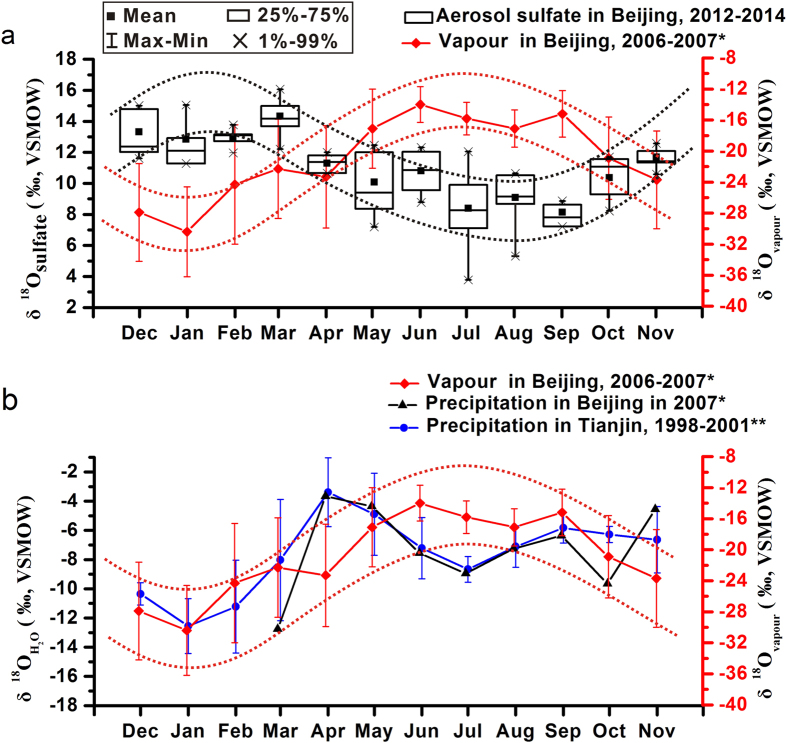
Relationship between δ^18^O_sulfate_ in aerosols and δ^18^O in atmospheric water vapor and precipitation^58^,^59^. The “*” represents the data from Wen *et al*. (2010)^58^; the “**” represents the data from GNIP Database^59^.

Evidence in particular for the latter, i.e. the increasing coal combustion in the winter, comes from the oxygen isotopic composition of sulfate aerosol (δ^18^O_sulfate_). It also exhibits strong seasonal changes, with the highest values in winter and low values in summer (Fig. 4a). Previous studies have shown that high-temperature combustion processes, thereby oxidizing the sulfur dioxide to sulfate, will lead to ^18^O enriched aerosol sulfate^25^,^33^, and δ^18^O_sulfate_ values of +35 to +40‰ have been reported^33^. Consequently, a higher contribution from coal combustion in the winter will cause more positive δ^18^O_sulfate_ values of sulfate aerosols. In addition, the lack of a positive correlation between the δ^18^O_sulfate_ and the δ^18^O_H2O_ (Fig. 7) supports the assumption that sulfate formed at high temperatures, rather than heterogeneous, i.e. aqueous oxidation of SO_2_ is the important process during the winter, because the latter would result in a positive correlation between δ^18^O_sulfate_ and the δ^18^O_H2O_^55^. This explains the obvious decoupling of both oxygen isotope records (Fig. 7) and argues that the observed increase in δ^18^O_sulfate_ seen in the winter is reflecting most likely a source effect, i.e. the high-temperature combustion of coal, generating an ^18^O enriched primary sulfate aerosol.

**Figure 8:**
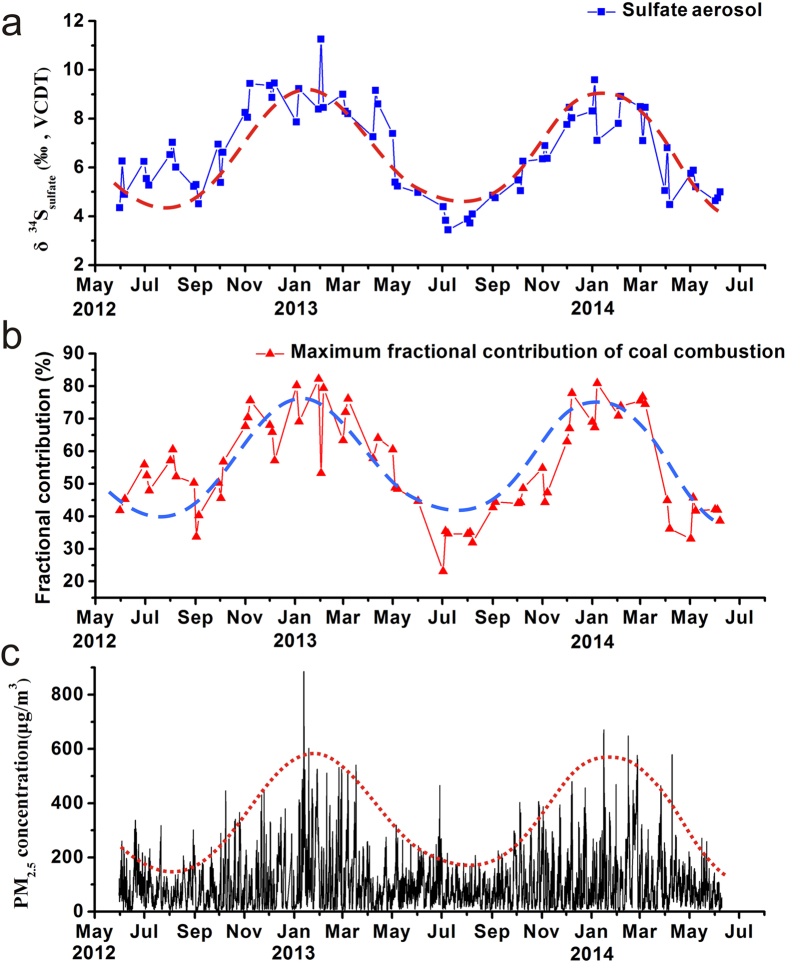
Variations in δ^34^S_sulfate_ values of sulfate aerosols, maximum contribution of coal combustion and PM_2.5_ concentration in Beijing, May 2012–June 2014. The data of PM_2.5_ concentration are from Beijing US Embassy^60^.

Although an influence of the meteorological boundary conditions, i.e. air temperature and atmospheric pressure, on the observed seasonality in the sulfur and oxygen isotope compositions of sulfate in Beijing aerosol cannot be ruled out, the tight coupling of the temporal trend in δ^34^S_sulfate_ and δ^18^O_sulfate_ is best explained as a variation related to the source of aerosol sulfate. Due to their pronounced seasonality, the two strongest variables in this respect are contributions from biogenic sulfur emissions, limited to the summer, and increasing coal consumption in the winter. In particular in the winter, coal combustion is the main contributor to the Beijing aerosol sulfate pool as evidenced by the paired shift to high δ^34^S and δ^18^O values for sulfate in aerosol. Moreover, the temporal variation in PM_2.5_ concentration during the sampling period exhibits an obvious seasonal trend (Fig. 8) that is similar to the temporal records of aerosol sulfate sulfur isotopic composition and the maximum contribution from coal combustion to the aerosol sulfate pool, i.e. high in winter and low in summer. This strongly underlines the conclusion that coal combustion is the major contributor to the Beijing aerosol (sulfate) pool. While the biogenic sulfur emissions are likely more difficult to control, a reduction in the usage of coal, especially the high sulfur coal, and the application of desulfurization measures for coal powered industries will be important in reducing sulfur emissions to the Beijing atmosphere, which will ultimately improve Beijing’s air quality.

## Methods

### Sample Collection

Total suspended particulates (TSP) were sampled on a 3-day basis from May 31, 2012 to June 10, 2014 (n = 73) on top of the roof (around 10 meters above ground level) of the Institute of Atmospheric Physics, Chinese Academy of Sciences, Beijing (Fig. 1). The samples were collected using a high volume air sampler (Qingdao Laoshan, KC1000) with a flow rate of 1.0 m^3^ min^−1^ and pre-combusted (450 °C for 6 h) quartz fiber filters (20 cm × 25 cm, Pallflex). After sampling, a pre-combusted glass jar (150 ml) with Teflon lined screw cap was used to store each filter in a freezer at −20 °C until geochemical analyses.

The meteorological data during sampling were obtained from China Meteorological Data Sharing Service System (http://cdc.nmic.cn/home.do, Fig. 2). The daily average values of air temperature, air humidity, wind speed and atmospheric pressure were calculated based on the observation data at 2.00 a.m., 8.00 a.m., 14.00 p.m. and 20.00 p.m. The detection limits of air temperature, precipitation, air humidity, wind speed and atmospheric pressure are 0.1 °C, 0.1 mm, 1%, 0.1 m/s and 0.1 hPa, respectively.

### Analytical Methods

Using a circular hole-puncher, two circular pieces with a diameter of 47 mm were cut from each filter (20 cm × 25 cm), shredded and soaked in 200 ml of Milli-Q water for 30 minutes added by ultrasonification^14^. Subsequently, the filters were kept in water overnight in order to quantitatively extract the water-soluble ions. The quartz filter fibers were removed by filtration using 0.45 mm millipore filters. 10 ml of the solution were taken for ion concentration analysis, and the remaining solution was acidified to pH<2 by addition of HCl solution and heated to boiling. The dissolved sulfate in the solution was precipitated as barite by adding 25 ml of 8.5% BaCl_2_ solution and the glass beaker with the solution was kept at 80 °C for additional 3 hours. The BaSO_4_ precipitates were collected on 0.22 μm acetate millipore filters and rinsed with 150 mL Milli-Q water to remove Cl^−15^. The millipore filters with the precipitates were dried in an oven at 45 °C for 48 hours. Then the S and O isotopic compositions of the BaSO_4_ precipitates were analyzed. The blank samples were also analyzed after the same method.

The concentrations of the water-soluble ions (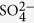, 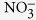, Cl^−^, F^−^, 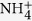, Na^+^, K^+^, Ca^2+^ and Mg^2+^) were analyzed by ion chromatography (Dionex ICS900). An IonPac^TM^ AS23 column (4 × 250 mm, Dionex) and an IonPac^TM^ CS12A column (4 × 250 mm, Dionex) were used f the determination of anions and cations, respectively. The 4.5 mM sodium carbonate and 0.8 mM sodium bicarbonate were used as the eluent for anions; the 20 mM methansulfonic acid (MSA) was used as the eluent for cations. The detection limits were below 0.07 μg/m^3^ for cations and anions.

The sulfur isotope measurements (δ^34^S) were performed at the Institute of Geographic Sciences and Natural Resources Research, Chinese Academy of Sciences, using an Elemental Analyzer (EA) coupled to a Delta V Advantage Isotope Ratio Mass Spectrometer (IRMS). Results are expressed in the standard delta notation relative to the Vienna Canyon Diablo Troilite standard (VCDT). The reproducibility of the measurements was better than ±0.2‰. The oxygen isotope values (δ^18^O) were measured by a Thermal Conversion Elemental Analyzer (TCEA) coupled to an IRMS. The results are reported in the standard delta notation against the Vienna Standard Mean Ocean Water (VSMOW). The reproducibility of the measurements was better than ±0.3‰.

### Sulfate Source Apportionment Calculation

Assuming that the seasonal change in δ^34^S_aerosol sulfate_ reflects temporal variations in the proportional contributions from different sulfate sources, these proportions can be estimated by using the following equations^16^,^20^,^49^:


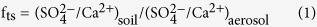


where the ratio of 
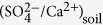
 is 0.18^50^;


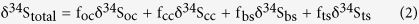



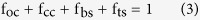


where f_oc_, f_cc_, f_bs_ and f_ts_ represent the fractional contributions of oil combustion, coal combustion, biogenic source and terrigenous source, respectively, and δ^34^S_oc_, δ^34^S_cc_, δ^34^S_bs_ and δ^34^S_ts_ represent the corresponding δ^34^S value of each sulfur source. We assume a δ^34^S_oc_ value of 20.5 ± 4.8‰^47^, a δ^34^S_cc_ value of 6.6 ± 3‰^47^,^48^, a δ^34^S_bs_ value of −6 ± 4‰^21^,^22^,^23^ and a δ^34^S_ts_ value of 4.5 ± 3.5‰^51^,^52^ as the respective δ^34^S signature of each sulfur source.

First, the fractional contribution from a terrigenous source has been calculated based on the equation (1) and the ratio of (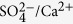) measured in aerosol.

Second, in winter, biogenic sulfur emissions are greatly attenuated to likely negligible since the soil microbial activity is weak. Hence, we assume that in winter, f_bs_ equals 0. Now, the fractional contributions from oil combustion and from coal combustion in winter can be calculated according to equations (2) and (3).

Third, the contribution from oil combustion is relatively constant throughout the year as there is no seasonal variation in oil consumption. Assuming that the average value of f_cc_ in winter equals the fractional contribution from oil combustion in spring, summer and autumn, the contributions of coal combustion and biogenic source in these seasons can also be computed.

## Additional Information

**How to cite this article**: Han, X. *et al*. Using stable isotopes to trace sources and formation processes of sulfate aerosols from Beijing, China. *Sci. Rep.* **6**, 29958; doi: 10.1038/srep29958 (2016).

## Figures and Tables

**Table 1:** Statistical parameters of stable isotopes (δ^34^S_sulfate_ and δ^18^O_sulfate_) and water-soluble ions in Beijing aerosols (SD represents standard deviation).

Species	Spring			Summer			Autumn			Winter		
	Range	Average	SD	Range	Average	SD	Range	Average	SD	Range	Average	SD
Na^+^(μg/m^3^)	0.4–1.8	1.1	0.5	0.1–1.0	0.5	0.2	0.2–2.1	1.1	0.6	0.7–4.4	2.0	1.1
NH_4_^+^(μg/m^3^)	0.6–38.5	9.9	12.6	0.3–25.3	7.4	7.0	1.8–14.0	7.5	3.7	1.4–23.8	8.8	5.9
K^+^(μg/m^3^)	0.4–3.6	1.6	1.1	0.2–2.8	1.0	0.6	0.7–3.8	1.8	0.8	0.4–3.9	1.6	1.0
Mg^2+^(μg/m^3^)	0.2–1.9	0.8	0.5	0.1–1.6	0.8	0.4	0.3–1.5	0.9	0.4	0.1–1.2	0.5	0.3
Ca^2+^(μg/m^3^)	4.7–24.9	11.3	6.9	3.1–10.7	6.8	2.3	3.4–14.8	9.8	3.7	1.5–13.8	6.2	3.1
F^−^(μg/m^3^)	0.1–2.1	0.7	0.6	0.0–0.5	0.3	0.2	0.3–1.3	0.6	0.3	0.1–1.0	0.5	0.3
Cl^−^(μg/m^3^)	0.6–6.6	2.8	1.7	0.2–3.8	1.0	0.8	0.3–11.1	4.3	3.1	1.7–12.4	6.5	3.5
NO_3_^−^(μg/m^3^)	2.3–89.8	30.7	32.7	2.5–54.6	19.6	13.9	9.3–43.4	25.7	12.2	2.7–46.3	16.3	12.6
SO_4_^2−^(μg/m^3^)	3.7–87.7	25.2	31.3	2.4–85.7	22.1	18.5	4.3–34.7	16.6	8.2	4.0–60.7	17.4	13.5
δ^34^S_sulfate_	4.5–9.2	6.4	1.5	3.4–7.0	5.0	0.9	5.0–9.4	6.8	1.3	7.1–11.3	8.6	0.9
δ^18^O_sulfate_	7.2–12.5	10.6	1.7	3.8–12.3	9.3	2.1	8.2–12.6	11.1	1.3	11.3–16.1	13.4	1.4

**Table 2:** Correlation coefficients for water-soluble ions and stable isotopes of atmospheric aerosol in Beijing (*Significant at 0.05 level; **Significant at 0.01 level).

	Na^+^	NH_4_^+^	K^+^	Ca^2+^	Mg^2+^	F^−^	Cl^−^	NO_3_^−^	SO_4_^2−^	δ^34^S	δ^18^O
Na^+^	1.00										
NH_4_^+^	0.29**	1.00									
K^+^	0.41**	0.72**	1.00								
Ca^2+^	0.14	0.64**	0.70**	1.00							
Mg^2+^	0.06	0.72**	0.68**	0.81**	1.00						
F^−^	0.47**	0.63**	0.73**	0.69**	0.47**	1.00					
Cl^−^	0.83**	0.39**	0.66**	0.31**	0.21*	0.66**	1.00				
NO_3_^−^	0.15	0.90**	0.72**	0.81**	0.84**	0.62**	0.26*	1.00			
SO_4_^2−^	0.16	0.93**	0.61**	0.63**	0.75**	0.54**	0.19	0.83**	1.00		
δ^34^S	0.66**	−0.01	0.26*	−0.17	−0.27*	0.34**	0.67**	−0.19	−0.13	1.00	
δ^18^O	0.55**	−0.04	0.27*	−0.15	−0.24	0.10	0.57**	−0.14	−0.26*	0.56**	1.00

**Table 3:** Relative contributions of sulfate sources to the sulfate in aerosols of Beijing (nd. represents no data).

Date	Sample	δ^34^S (‰)	δ^18^O (‰)	Contribution of terrigenous sulfate (%)	Contribution of biogenic sulfur (%)		Contribution of coal combustion (%)		Contribution of oil combustion (%)	
					Min.	Max.	Min.	Max.	Min.	Max.
2012/5/31	Fu30	4.3	11.5	4.0	30.5	50.7	33.7	41.8	11.5	23.7
2012/6/6	Fu32	4.9	12.1	5.1	26.0	46.9	36.6	45.3	11.5	23.7
2012/6/30	Fu42	6.2	3.8	5.2	15.3	38.2	45.1	55.9	11.5	23.7
2012/7/3	Fu43	5.5	7.1	2.6	21.3	43.5	42.4	52.5	11.5	23.7
2012/7/6	Fu44	5.3	9.9	5.5	22.9	44.3	38.7	47.9	11.5	23.7
2012/8/1	Fu54	6.5	10.7	6.3	12.9	36.0	46.2	57.1	11.5	23.7
2012/8/4	Fu55	7.0	5.3	7.1	8.8	32.5	48.8	60.5	11.5	23.7
2012/8/8	Fu56	6.0	8.7	7.4	16.8	38.9	42.1	52.2	11.5	23.7
2012/8/30	Fu63	5.2	8.9	2.2	23.9	45.7	40.6	50.3	11.5	23.7
2012/9/2	Fu64	5.3	nd.	22.7	19.9	38.5	27.2	33.7	11.5	23.7
2012/9/5	Fu66	4.5	7.8	7.4	28.7	48.6	32.5	40.3	11.5	23.7
2012/9/29	Fu75	7.0	nd.	18.7	7.4	29.2	40.6	50.3	11.5	23.7
2012/10/2	Fu76	5.4	8.2	9.2	21.5	42.4	36.9	45.6	11.5	23.7
2012/10/5	Fu77	6.6	9.3	7.7	11.9	34.9	45.8	56.8	11.5	23.7
2012/11/1	Fu86	8.3	12.1	10.1	0.0	23.7	54.7	67.7	11.5	23.7
2012/11/4	Fu87	8.0	10.6	5.0	1.0	26.7	56.9	70.4	11.5	23.7
2012/11/7	Fu88	9.4	11.4	12.0	0.0	15.5	61.1	75.6	11.5	23.7
2012/12/1	Fu96	9.4	12.4	5.4	0.0	0.0	54.9	68.0	15.3	31.5
2012/12/4	Fu97	8.9	12.0	11.0	0.0	0.0	53.2	65.9	13.3	27.4
2012/12/7	Fu98	9.5	14.8	13.8	0.0	0.0	46.1	57.1	16.8	34.6
2013/1/3	Fu107	7.9	nd.	6.8	0.0	0.0	64.8	80.2	7.5	15.4
2013/1/6	Fu108	9.2	15.1	5.6	0.0	0.0	55.8	69.1	14.7	30.1
2013/1/30	Fu116	8.4	13.1	1.1	0.0	0.0	66.4	82.2	9.7	19.9
2013/2/2	Fu117	11.3	13.2	3.1	0.0	0.0	43.0	53.3	25.3	51.9
2013/2/5	Fu118	8.5	12.7	2.9	0.0	0.0	64.1	79.4	10.2	21.0
2013/3/1	Fu127	9.0	14.2	12.2	0.0	0.0	51.1	63.3	14.2	29.2
2013/3/4	Fu128	8.3	15.0	10.3	0.0	0.0	58.2	72.0	10.2	21.0
2013/3/7	Fu129	8.2	16.1	7.6	0.0	0.0	61.5	76.1	9.4	19.4
2013/4/7	Fu141	7.3	nd.	12.6	6.0	29.3	46.6	57.8	11.5	23.7
2013/4/13	Fu144	8.6	10.7	17.9	0.0	18.9	51.7	64.0	11.5	23.7
2013/5/1	Fu155	7.4	8.4	10.6	5.3	29.1	48.9	60.5	11.5	23.7
2013/5/4	Fu156	5.4	9.4	5.6	21.9	43.5	39.4	48.8	11.5	23.7
2013/5/7	Fu157	5.2	7.2	4.5	23.5	44.9	39.1	48.4	11.5	23.7
2013/6/1	Fu169	5.0	10.7	6.5	25.1	45.9	36.1	44.7	11.5	23.7
2013/7/2	Fu331	4.4	9.2	26.8	26.4	43.0	18.7	23.1	11.5	23.7
2013/7/5	Fu332	3.8	8.3	6.7	34.2	53.2	28.6	35.4	11.5	23.7
2013/7/8	Fu333	3.4	12.1	3.9	37.8	56.6	28.0	34.7	11.5	23.7
2013/8/1	Fu341	3.9	10.5	8.3	33.5	52.3	27.9	34.5	11.5	23.7
2013/8/4	Fu342	3.7	10.2	6.1	35.1	54.0	28.4	35.1	11.5	23.7
2013/8/7	Fu343	4.1	9.2	13.4	31.0	49.3	25.8	31.9	11.5	23.7
2013/9/1	Fu352	4.9	7.2	7.8	25.9	46.2	34.5	42.7	11.5	23.7
2013/9/4	Fu353	4.8	8.6	4.8	27.2	47.8	35.8	44.4	11.5	23.7
2013/10/2	Fu362	5.5	11.6	12.1	20.2	40.8	35.6	44.0	11.5	23.7
2013/10/5	Fu363	5.0	11.1	7.7	24.4	45.0	35.8	44.3	11.5	23.7
2013/10/8	Fu364	6.3	11.7	14.0	13.7	35.2	39.3	48.6	11.5	23.7
2013/11/1	Fu373	6.3	11.4	7.4	14.1	36.8	44.3	54.8	11.5	23.7
2013/11/4	Fu374	6.9	12.1	25.3	6.8	27.4	35.7	44.3	11.5	23.7
2013/11/7	Fu375	6.4	12.6	16.7	12.4	33.6	38.2	47.3	11.5	23.7
2013/12/1	Fu383	7.8	15.0	22.1	0.0	0.0	50.9	63.0	8.7	17.8
2013/12/4	Fu384	8.5	14.1	13.3	0.0	0.0	54.1	67.0	11.4	23.5
2013/12/7	Fu385	8.0	11.6	7.5	0.0	0.0	62.9	77.8	8.5	17.4
2014/1/1	Fu393	8.3	12.9	12.7	0.0	0.0	55.7	69.0	10.6	21.8
2014/1/4	Fu394	9.6	12.1	4.3	0.0	0.0	54.4	67.3	16.5	33.8
2014/1/7	Fu395	7.1	11.3	12.1	0.0	0.0	65.3	80.9	4.1	8.4
2014/2/2	Fu403	7.8	12.0	15.1	0.0	0.0	57.2	70.9	8.1	16.7
2014/2/5	Fu404	8.9	13.8	4.1	0.0	0.0	59.6	73.8	12.8	26.3
2014/3/1	Fu411	8.5	14.8	5.9	0.0	0.0	61.0	75.5	10.7	22.1
2014/3/4	Fu412	7.1	12.2	15.7	0.0	0.0	61.9	76.7	4.4	9.0
2014/3/7	Fu413	8.5	13.7	6.9	0.0	0.0	60.2	74.5	10.7	22.0
2014/4/3	Fu422	6.8	10.7	23.7	7.7	28.5	36.3	44.9	11.5	23.7
2014/4/6	Fu423	4.5	11.4	12.0	28.1	47.2	29.2	36.2	11.5	23.7
2014/5/2	Fu432	5.8	12.0	27.9	15.4	33.9	26.7	33.1	11.5	23.7
2014/5/5	Fu433	5.9	12.5	14.0	16.7	37.6	36.9	45.7	11.5	23.7
2014/5/8	Fu434	5.2	11.1	12.3	22.3	42.5	33.7	41.7	11.5	23.7
2014/6/1	Fu441	4.6	9.6	6.4	27.9	48.1	34.0	42.1	11.5	23.7
2014/6/4	Fu443	4.8	8.8	7.7	26.7	46.9	33.9	42.0	11.5	23.7
2014/6/7	Fu444	5.0	12.3	14.1	23.7	43.3	31.1	38.6	11.5	23.7
